# Lymphangiogenesis and Lymphatic Barrier Dysfunction in Renal Fibrosis

**DOI:** 10.3390/ijms23136970

**Published:** 2022-06-23

**Authors:** Jing Liu, Chen Yu

**Affiliations:** Department of Nephrology, School of Medicine, Tongji Hospital, Tongji University, Shanghai 200065, China; liujing961226@163.com

**Keywords:** lymphatic system, permeability, lymphangiogenesis, renal fibrosis

## Abstract

As an integral part of the vascular system, the lymphatic vasculature is essential for tissue fluid homeostasis, nutritional lipid assimilation and immune regulation. The composition of the lymphatic vasculature includes fluid-absorbing initial lymphatic vessels (LVs), transporting collecting vessels and anti-regurgitation valves. Although, in recent decades, research has drastically enlightened our view of LVs, investigations of initial LVs, also known as lymphatic capillaries, have been stagnant due to technical limitations. In the kidney, the lymphatic vasculature mainly presents in the cortex, keeping the local balance of fluid, solutes and immune cells. The contribution of renal LVs to various forms of pathology, especially chronic kidney diseases, has been addressed in previous studies, however with diverging and inconclusive results. In this review, we discuss the most recent advances in the proliferation and permeability of lymphatic capillaries as well as their influencing factors. Novel technologies to visualize and measure LVs function are described. Then, we highlight the role of the lymphatic network in renal fibrosis and the crosstalk between kidney and other organs, such as gut and heart.

## 1. Introduction

As a component of the cardiovascular system, the lymphatic vasculature absorbs and transports extravasated fluid and macromolecules back to the blood circulation, and therefore maintains tissue homeostasis. Moreover, the lymphatic system is important for lipid transportation, immune surveillance, etc. [1]. The lymphatic vasculature consists of lymphatic capillaries (also called initial lymphatic vessels (LVs)), collecting LVs and valves that work together to keep fluid balance [2,3]. Although they belong to the same system, initial LVs and collecting LVs have different structures, corresponding to their distinct functions [4]. The single layer of loosely connected lymphatic endothelial cells (LECs) with discontinuous button-like intercellular junctions enable the easy uptake of interstitial materials in initial LVs, while the continuous zipper-like tight junction of collecting LVs and the surrounding layer of smooth muscle cells prevent extravasation of lymph during transport. Whereas the function of blood vessels has been well established, our understanding of LVs, especially lymphatic capillaries (the focus of this paper is on permeability and proliferation), biology and pathology remain ambiguous.

Fibrosis, which can occur in almost all organs, is responsible for most organ failures. Although its regression has been reported in some organs, such as liver [5], fibrosis remains difficult to reverse in many other organs. Repetitive or severe injury in chronic inflammatory disorders results in an abnormal tissue repair response, characterized by overaccumulation of collagen and fibronectin. Data from previous research have established that organ fibrosis and abnormalities of its own lymphatic system appear to be closely linked [6,7]. For digestive system, LVs intensify the trafficking of toxins from the gut to the systemic blood, enhancing immune reaction of the host, and thus contribute to the development of liver cirrhosis [8]. The increased number of LVs in heart appears to be beneficial in fibrosis of the cardiovascular system. Enhanced proliferation and function of LVs alleviates fibrosis and preserves cardiac function by reducing interstitial fluid pressure as well as accelerating resolution of inflammation in myocardial infarction [9]. The lymphoangiocrine function of LECs in repairing cardiac injury has also been reported [10]. However, unlike the clear role of the lymphatic system in heart and liver diseases, the function of LVs in renal fibrosis is a field in its infancy and has inconsistent results.

In this review, we aim to discuss recent advances in our understanding about two main properties (permeability and proliferation) of initial LVs, and then put special focus on the lymphatic network within the context of renal fibrosis. How renal function is closely linked to intestinal and cardiac performance through the lymphatic system is also addressed. Additionally, the lack of techniques for measurement and visualization has been a major contributing factor to the failure in studying development and function of the lymphatic vasculature; therefore, we also review the current and emerging approaches in lymphatic-implicated research.

## 2. Permeability

LVs start with the blind-ended lymphatic capillaries in almost all organs, removing not only unwanted byproducts, but also taking away circulating substances such as fluid, cells and, in the case of mesenteric LVs, lipids. Thin endothelial walls, overlapping arrangement and button-type intercellular junction of initial LVs allow fluid and solutes to enter the tubes easily, therefore forming lymph [11]. Immune cells, fluid, immune cells, lipids, macromolecules and antigens are collectively known as lymph. There are two routes related to lymph formation described by previous research, namely paracellular route and transcellular route.

**Figure 1:**
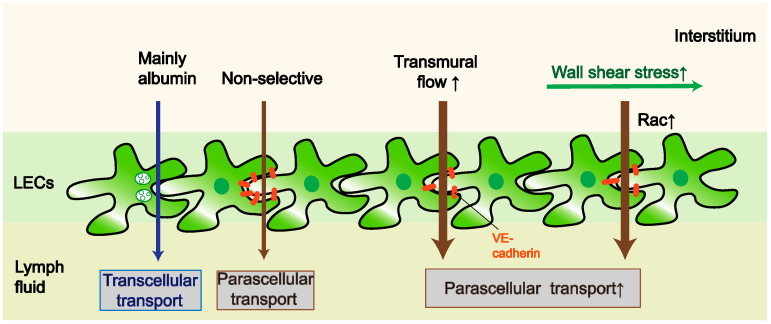
**Transportation through lymphatic endothelial cells.** Solutes are mainly transported indiscriminately via the paracellular pathway (brown arrow); the transcellular pathway partly transports albumin through formation and release of vesicles (blue arrow); transmural flow and wall shear stress increase endothelial cell permeability through tight junction protein and Rac, respectively. LEC: lymphatic endothelial cell.

As mentioned above, discontinuous intercellular junction protein expression of LECs renders high permeability to the walls of initial LVs. For long, this pathway of cell–cell junction, in coordination with anchoring filaments and obeying the Starling law [12] to form lymph was considered as the sole route. However, recent research is challenging this traditional dogma and proposing a completely new lymph-generation mechanism: vesicular transport, also called transcellular route [13]. The abundance of vesicles inside LECs have been proved with electron microscopy decades ago [14], and tracer particles injected into the interstitial compartment were detected in vesicles of LECs [15]. By measuring albumin uptake, Triacca V et al. [13] showed that LECs actively uptake solutes through vesicle formation and transcytosis. Additionally, their former studies found increased effective permeability of LECs due to transmural flow was lost after blocking the transcellular-related pathway [16]. However, their in vitro model only based on cultured cells, limiting the application of these results to in vivo experiments. Last year, Jannaway M et al. [17] addressed this limitation by measuring the solute flux from single collecting LVs in mice. They pointed out the difference in solute transported by para-endothelial and transendothelial pathways, with paracellular route transport without selectivity, while vesicles specifically uptake bovine serum albumin (BSA) (Figure 1).

### 2.1. Factors That Affect the Paracellular Pathway

For endothelial cells, cell junction molecules (such as vascular endothelial-Cadherin (VE-Cadherin), which is particularly enriched in button junctions) are responsible for cell–cell interactions, and integrins mediate interaction between endothelial cells and extra cellular matrix [18]. These two mediators are connected with cytoskeletal machinery and several signaling molecules when facing the mechanical signals such as shear stress (SS) or stretch. Among all mechanical signals, SS is the most studied stimuli. There are two kinds of SS in vessels. Laminar shear stress (LSS) exists in straight long vessels, and oscillatory SS is generated when fluid is disturbed at branch points [19]. Barrier function of the lymphatic endothelium increases with enhanced laminar flow [20,21], mechanism of which may be related to the small GTPase Rac1, which directs cytoskeletal reorganization at intercellular junctions [20]. Collagen fibers of LECs are attached to anchoring filaments, which can be pulled apart by increased interstitial pressure, resulting in opening of the LV lumen and enhancing uptake of lymph components [22]. After a drop in interstitial fluid pressure, the anchoring wire springs back to close this permeable channel. Most LVs of skin are relatively collapsed in shape under physiological condition [23], and they are pulled open by anchoring filaments under pathological condition [24]. However, overextension of LVs leads to the impairment of barrier and leakage [25]. This paracellular fluid transportation in lymphatic endothelium generates transmural SS. Decreased expression of tight junction, in the presence of elevated transmural SS, leads to increased permeability of endothelium [16,21]. Since transmural SS can help recruit CCR7^+^ immune cells into LVs and lymph nodes via promoting expression of cytokine CCL21 expression in LECs, it has been regarded as an important regulator of LEC function as well as an inflammatory cue at early stage for lymphatics [16]. Reduced transmural SS caused by lymphatic drainage stagnation in secondary lymphedema or experimental lymphedema mode reduced dendritic cells transmigration into lymph [16].

In addition to SS, extensive research has shown that inflammatory cytokines such as tumor necrosis factor-α (TNF-α), interleukin (IL)-1β, lipopolysaccharides, C-reactive protein change the permeability of solutes [26]. LVs and inflammation are inextricably linked. On one hand, LVs are responsible for uptake and transport of immune cells from tissues to lymph nodes for following inflammatory response, and on the other hand, inflammatory factors influence the function of LVs, including vascular permeability and pumping activity [27]. After synthesizing and assembling hyaluronic acid glycocalyx on the surface, immune cells can provide the hyaluronan by themselves to adhere lymphatic vessel endothelial protein(LYVE-1), therefore docking to LECs [28]. Although there is extensive evidence that hyaluronic acid influences endothelial permeability, most of them are based on vascular endothelium [29,30,31], not lymphatic endothelium. Increased permeability of cultured rat LECs after IL-6, TNF-α, and interferon-γ treatment was blocked or reduced by N(G)-nitro-L-arginine methyl ester, suggesting a regulator role of nitric oxide (NO). Possible mechanisms involve the loss of tight junction protein, VE-Cadherin [26]. Although a clear picture of the mechanisms underlying immune-induced LV barrier disruption has not emerged, Kajiya K identified elevated vascular endothelial growth factor(VEGF)-A as a possible mechanism to explain the impaired LV permeability induced by ultraviolet B [32]. VEGF-A increases in plasma and tissues of multiple inflammatory diseases [33]. Additionally, both VEGF-A transgenic mice and mice that underwent adenoviral delivery of murine VEGF-A164 gene had enlarged but nonfunctional LVs, indicating a potential link between VEGF-A and LV barriers [34]. Mast cells are major secretory cells of the innate immune system. Enhanced synthesizing and secreting ability of mast cells not only promote migration of immune cells but also affect lymphatic permeability [35]. The modulators influencing vessel permeability include histamine, leukotrienes, VEGF, bradykinin, and interferon-γ [36]. In addition to the loss of tight junction protein, transformation of tight junction was also reported in a number of inflammatory diseases [4,37]. In chronic inflammation states, intercellular junctions of LECs transformed from buttons to zippers, which gradually back to normal after inflammation resolution [37]. However, the direct mechanical link between inflammation and “button-to-zipper” transition of LECs is still not established. Moreover, in the sustained alcohol-induced immune disorder model, altered permeability of LECs was not associated with decreased tight junction protein expression, calling for further investigation into the localization of junction protein to better understand other possible mechanisms of LEC permeability [38]. Impaired LEC barrier function may lead to two consequences. One is the less efficient fluid clearance, which exacerbates systemic edema. The other is the leakage of lymph, causing accumulation of inflammatory mediators in tissues and therefore enhances the local inflammation as well as the development of fibrosis.

### 2.2. Factors That Affect the Transcellular Pathway

Although the passive, paracellular transport has been identified as the predominant route of macromolecular solute entry, the active, transcellular pathway also plays a non-negligible role in lymph formation. As a critical part of endocytosis and exocytosis of transcellular route in LECs, caveolae abnormalities always mediate dysfunction of transport. Mice constitutively lacking Caveolin-1, the most abundant isoform of Caveolin, had significantly higher initial fluid pressure, albumin extravasation and collagen content. These results were similar in mice with LEC-specific gene deletion [39]. In addition, caveolae also impacts collecting LVs through regulating NO, altering the location and activity of connexins as well as calcium channels such as transient receptor potential vanilloid-4 [40].

Although not well studied, the effect of SS on transcellular pathways has been mentioned in recent years. Changes in transmural pressure generated by the paracellular route also can regulate transendothelial route, suggesting coordination of two lymph-generating pathways [13]. Additionally, abnormally high LSS (20 dynes/cm^2^) promotes differentiation of LVs to veins [41], implying that LECs may lose their identity under non-physiological SS.

### 2.3. Outstanding Questions Related to LV Permeability

As is often the case with recent studies that provide answers to understand questions published decades ago, they also invite to raise questions. First, as mentioned earlier, sustained inflammation may lead to two opposite outcomes: increased permeability due to loss of button proteins and decreased permeability mediated by button-to-zipper transition, and further work in this area is warranted to understand which one prevails in disease states. Secondly, previous studies about transcellular route have either used cultured cells [13] or single location of LVs [17], yet the permeability of LVs to solutes may be tissue-specific. Therefore, future studies about the uptake of BSA and other solutes in LVs from different tissues are suggested. Thirdly, due to technical limitations and difficulty of detection, initial LVs were often replaced by collecting LVs in studies, making it difficult to interpret the results in the lymphatic capillaries. However, it is initial LVs, not secondary LVs that uptake solutions in tissues. To develop a full picture of initial LVs, additional studies will be needed with new techniques that detects lymphatic vascular permeability. Fourthly, as discussed in an excellent comment covered by Kalucka J et al. [42], in addition to BSA, do other macromolecules such as immunoglobulins also transport with the transcellular pathway, thereby potentially enhancing antigen delivery to lymph nodes under inflammatory conditions? If such transcellular pathway-mediated macromolecular transport exists, are specific receptors required to mediate transcytosis? Metabolic pathways LECs use to generate energy for vesicles transport is also an important issue for future research. LECs may utilize different pathways from vascular endothelial cells since the disparity between nutrient availability in blood and lymph.

## 3. Proliferation

The production of the lymphatic network is as important as vascular vessels during human development. When the lymphatic system has been established, LECs remain stationary and back to proliferation and differentiation after receiving stimulation [43]. The proliferation of LVs, also called lymphangiogenesis, occurs not only physiologically during wound healing and corpus luteum development, but also pathologically in inflammation, tumor and transplant rejection [44,45,46]. Determining the regulators of lymphatic proliferation is the basis of treating pathological lymphangiogenesis.

### 3.1. SS

SS produced by fluid flow is a vital regulator of LVs formation both during physical development and pathological conditions. Lymphangiogenesis starts with sprouting and filopodia formation at the growing front of LECs via stimulating VEGF-C/VEGF receptor (VEGFR)-3 signaling. Before lumenization, these newborn LECs are not exposed to lymphatic fluids or fluid-generated LSS. When the mature lumens are formed, SS caused by lymph promotes tip cells become quiescent [43]. The precise mechanisms that LSS inhibiting LECs sprouting are not fully understood, with reports suggesting either downregulation of delta-like 4 accompanied with upregulation of VEGF-C signaling [43], Krüppel-like factor-dependent activation of highly selective calcium channel ORAI1 [47], or suppressing Notch activity [48].

### 3.2. Inflammation

**Figure 2:**
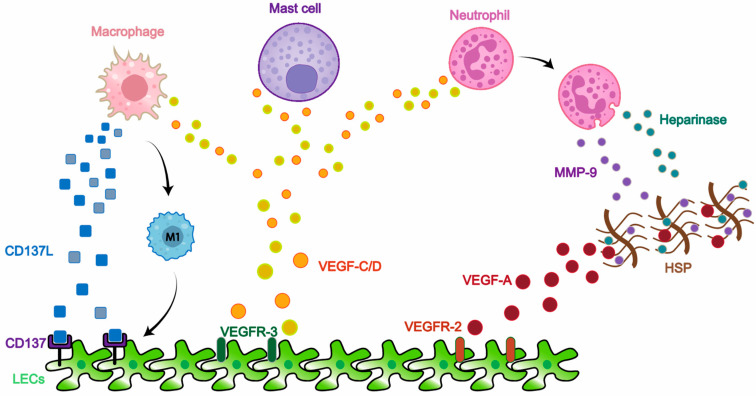
**Schematic diagram of immune cells contributing to lymphatic proliferation.** Macrophages promote lymphangiogenesis through: ① secreting CD137 ligand to bind with CD137 expressed on LECs; ② differentiating into M1-type macrophages and then into LECs; ③ secreting VEGF-C and VEGF-D in inflammation which binds with VEGFR-3 on LECs. Mast cells promotes lymphatic proliferation through producing VEGF-C and -D. Neutrophils participate the lymphangiogenesis process not only through releasing VEGF-C/D but also secreting MMP-9 and heparinase, which release VEGF-A from HSP and increase the bioactivity of VEGF-A. HSP: heparan sulphate preoteoglycan; LEC: lymphatic endothelial cell; MMP: matrix metalloproteinase; VEGF: vascular endothelial growth factor; VEGFR: vascular endothelial growth factor receptor.

Lymphangiogenesis can also be mediated by the cooperation of immune cells (Figure 2) since many inflammatory diseases are accompanied by enhanced lymphatic growth [49].

Macrophages are of critical importance, as proved in peritonitis mice models, where CD11B^+^ macrophage-derived VEGF-C/D may be mediators of increased LVs density and sprouts [50]. Transgenic VEGF-C overexpression leads to augmentation and expansion of the lymphatic network [51,52]. VEGF-C, which was released by macrophages efferocytosis in latest report of Gilnton et al., inhibited proinflammatory cytokine production in an autocrine fashion and promoted lymphangiogenesis in a paracrine manner, thereby facilitating recovery from myocardial infarction [53]. Although the action of VEGF-C/D on VEGFR-3 is the most studied pro-lymphangiogenic pathway [54], evidence has emerged for a vital involvement of other VEGF family factors in lymphatic proliferation. VEGF-A/VEGFR-2, an essential hemangiogenic signaling route, also plays a potential role in lymphangiogenesis through recruitment of macrophages in inflammation [34,55,56]. In addition to VEGF, there are many additional factors released by macrophages at play. Binding of CD137 expressed on hypoxic LECs and CD137 ligand secreted by macrophages promotes growth of LVs in unilateral ureteral obstruction (UUO) mice [57]. Moreover, the close relationship between macrophages and LECs also contributes to lymphangiogenesis. Zhang Y et al. suggested that VEGF-C/VEGFR-3 suppressed macrophage autophagy thereby promoting their differentiation into M1 macrophages and further transdifferentiation of M1 into LECs [58]. As the largest manifestation of inflammatory changes involving LVs, macrophages have a greater function in studies of lymphoedema. Macrophages recruitment immediately exists after lymphatic injury, implying multiple roles of macrophages in lymphoedema [59]. VEGF-C-mediated lymphangiogenesis alleviates fluid accumulation after surgery [60]; however, strong expression of iNOS by macrophages injures LVs function through attenuating lymphatic pumping [61].

Neutrophils are of equal importance. At the site of inflammation, neutrophils infiltration appeared around lymphatic microvessels before proliferation of the lymphatic network [62]. Similar to macrophages, neutrophils contribute to lymphangiogenesis mainly by secreting molecules in inflammation. Most VEGF-A isoforms bind to extracellular matrix, but only part of secreted VEGF-A has bioactivity [63]. Neutrophil-derived matrix metalloproteinases 9 and heparinase work together to liberate VEGF-A from matrix and raise VEGF-A biological activity, thereby coordinating lymphatic growth [64]. Additionally, neutrophils act as organizers of lymphangiogenesis via releasing VEGF-D, albeit to a lesser extent [64]. Furthermore, neutrophils, cooperating with macrophages, contributed to inflammatory bowel disease-related lymphangiogenesis by producing VEGF-A, VEGF-C and VEGF-D in zebrafish [65]. Lymph nodes have a similar VEGF-mediated lymphangiogenic pattern to tissues, and neutrophils compensate for the generation of VEGF and thus support lymphangiogenesis in mice lacking B cells [64].

The role of other immune cells has been far less explored than macrophages and neutrophils. The evidence that cultured human mast cells produce lymphangiogenic molecules VEGF-C/D to combine with VEGF-R1/2 [66] established a possible connection between mast cells and lymphangiogenesis. Actually, in patients with breast cancer, mast cell density was correlated with LV number inside and around tumors [67].

## 4. Technologies of Lymphatic Research on Imaging and Function

Due to the few contents of lymphatic fluid, it is difficult to locate and cannulate LVs with angiographic techniques. Clinical methods to visualize LVs include lymphoscintigraphy, magnetic resonance lymphography, PET/CT, contrast-enhanced ultrasound and near-infrared fluorescence imaging; however, they are bound by several limitations [68]. For animal models of lymphatic function, immunostaining for antibodies against LEC markers (prospero homeobox protein 1 (Prox1), VEGFR-3, LYVE1, and podoplanin) in tissue sections is the most common method. Combination of these markers is recommended since they are not exclusive to LVs. However, this standard two-dimensional method may not accurately quantify changes in LVs that occur at the organ level.

Digital three-dimensional (3D) reconstructions provide far more detailed information of lymphatic capillaries than traditional tissue sections [69,70]. Using whole-mount immunolabel of isolated organs with LEC markers, in conjunction with optical clearance and imaging with confocal microscopy, 3D images of intact tissues are obtained [71]. Hägerling et al. rapidly identified heterogeneity between skin samples of healthy control and diseases with volume information-based histopathological analysis by 3D reconstruction and data extraction (VIPAR) and found disrupted LVs in patients with lymphoedema [70]. Lymphatic extravasation, which may be caused by disruption of LVs, facilitated edema development in clinical settings. Therefore, quantitative analytic approaches, such as VIPAR, can be used as an early warning method before the onset of clinical symptoms. Due to the experimental advantage of 3D reconstruction techniques in identifying newly formed non-functional lymphatic segments, it has also been used to study the spatiotemporal dynamics and differences of LVs in development and pathological states [69,71].

Specific labeling of LVs with fluorescent dyes also partially compensates for the shortcomings of conventional immunohistochemistry [72]. To further elucidate important lymphatic formation and transport, transgenic mouse lines based on gene-targeted bacterial artificial chromosome and expressing either mOrange, tdTomato or green fluorescent protein under Prox1 transcriptional control have been reported in previous studies [73]. The design of dual fluorescent transgenic mice made it possible to visualize LVs in the context of hemangiogenesis [74]. Recently, combination of indocyanine green (ICG) and near-infrared fluorescent (NIRF) provided deeper light penetration in non-invasive optical imaging of LVs in vivo [75]. ICG/NIRF technique has been performed clinically to characterize defects of lymphatic transport or proliferation in patients with lymphedema [76].

In addition to in vivo experiments, isolation and cannulation of LVs in ex vivo experiments have been utilized when study contractile behavior such as contraction frequency, amplitude and ejection fraction [77]. Moreover, ex vivo studies allow for greater control of experimental conditions [68] (Table 1).

## 5. LVs and Renal Fibrosis

### 5.1. The Renal Lymphatic System

Although the anatomy of the kidney lymphatic system was described several decades ago [78] and cortical LVs have been identified in various species, whether LVs exist in renal medulla is still contentious. There are two lymphatic systems responsible for the drainage of cortical lymphatic fluid. Beginning as the blind-ended lymphatic capillaries in cortex, the first lymphatic system runs adjacent to arcuate arteries to the corticomedullary junction. Accompanied by interlobular vessels, these initial LVs drain into the renal pelvis, hilar lymphatics, renal draining lymph nodes and eventually, systemic circulation [79]. The second renal lymphatic system is more superficial with limited function of draining cortical parts. As conduits for the drainage of glomerulus-filtered fluid, LVs, tubules and peritubular capillaries maintain intercortical pressure. Several lines of early evidence suggest that venous pressure and solute load in the interstitium appear to be related to renal lymph flow [80]. The composition of the renal lymphatic fluid corresponds to the function of kidney. As reflected in the hilar lymph, renal lymph includes immunoglobulins, albumins, coagulation factors, cellular apoptosis-related factors, electrolytes, immune cytokines, renin and angiotensin II [81]. The drainage and composition of lymphatic fluid are altered accordingly in states of renal diseases.

Abnormalities of renal lymphatic systems, including proliferation and dysfunction, are associated with conditions such as acute kidney injury, polycystic kidney disease, transplantation rejection and peritoneal ultrafiltration failure (reviewed in Donnan et al. [82]). Renal fibrosis, which is the consequence of pathological accumulation of extracellular matrix, is a common feature of almost all chronic kidney diseases (CKD). The results based on human and animal disease models have established a link between the renal lymphatic system and fibrosis [83,84], but mechanisms have yet to be elucidated.

### 5.2. Lymphatic Proliferation and Renal Fibrosis

Renal LVs deliver fluid, protein and soluble antigens from kidney to renal lymph nodes, and then to systemic network [85]. Lymph, especially lipid and protein, retention in kidney induced by renal lymphatic ducts legation could damage tubular epithelial cells(TECs) and generate fibrogenic mediators [86]. Therefore, renal fibrosis characterized by TECs apoptosis and collagen deposition occurred in the late stage of lymph circulation disturbance [87].

The origin of neoplastic LECs is still debatable. Although it has been a generally accepted conception that most new LVs originate from intrinsic tissue LECs [88], emerging evidences have suggested the involvement of bone marrow-derived cells in LVs formation. The Y chromosome was detected in de novo LECs from male kidney transplant recipients with female donors, implying that circulation may be a potential origins of lymphatic progenitor cells [89]. Actually, Lee YJ et al. has recognized bone marrow-derived podoplanin-positive macrophages as progenitor cells of LECs in wound and tumor models [90]. In addition to macrophages, Hur J et al. proved that podoplanin-positive monocytes promoted lymphatic neovascularization after interacting with platelets that had C-type lectin-like receptor-2, a receptor of podoplanin [91]. These interesting studies not only provide a new pathway for LVs proliferation, but also expand the role of podoplanin from a LECs marker to an active regulator of lymphangiogenesis. However, former studies have failed to address on relationship of podoplanin and lymphangiogenesis in kidney diseases. In peritonitis model, elevated podoplanin expression accelerated actin remodeling and promoted macrophage trafficking toward CCL21, which is highly expressed on new-born LVs [92]. Therefore, there is abundant room for further progress in determining origin of injury-related lymphangiogenesis and subtypes of circulation-derived lymphatic progenitor cells.

**Figure 3:**
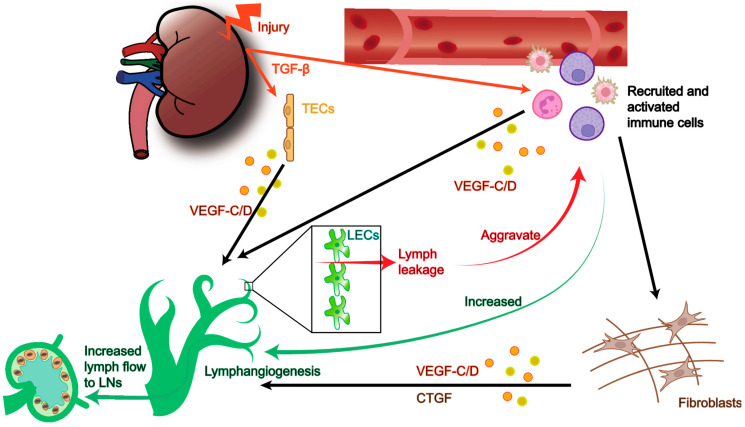
**Lymphatic vascular proliferation in chronic kidney injury.** In CKD, stimulated by TGF-β, TECs and activated immune cells (including macrophages, neutrophils, dendritic cells, and lymphocytes) are main sources of growth factors such as VEGF-C/D and CTGF, thereby promote lymphangiogenesis (black arrow). Expansion of the lymphatic network has a dual impact on CKD. The positive effect is that increased LVs promote the clearance from inflamed tissues (green arrow), while the negative influence is that hyperpermeability of injury-generated LVs may lead to lymph leakage and thus aggravate immune cytokines accumulation in kidney (red arrow). CKD: chronic kidney disease; CTGF: connective tissue growth factor; LEC: lymphatic endothelial cell; LN: lymph node; TEC: tubular epithelial cell; VEGF: vascular endothelial growth factor.

In kidney, tubule cells [32] and infiltrating macrophages [93] are regarded as two main sources of lymphangiogenic factor VEGF-C. Active lymphangiogenesis at the site of renal interstitial lesions develops in different fibrosis models [84,94] and CKD patients with multiple pathologies [83]. To date, although the pathophysiological relevance of this strong association remains controversial, dual effects of lymphangiogenesis on renal fibrosis development are widely accepted. The beneficial influence is that proliferated LVs assist removal of inflammatory cytokines from injured tissues, moderating tubular epithelial damage and fibrosis progression [82,95]. LVs proliferation, which was promoted by recombinant human VEGF-C, attenuated renal fibrosis with reduced infiltrating macrophages and level of transforming growth factor β1 (TGFβ1) in UUO mice [95]. However, other research supports a detrimental role for LVs formation in the setting of chronic kidney injury. Like pre-existing LVs, newly generated LVs also express CCL21, which enhances the recruitment of CCR7^+^ immune cells into renal draining lymph nodes and spleen, thereby aggravating systemic inflammation and renal fibrosis [94]. Inhibition of either lymphangiogenesis or CCR7^+^ dendritic cells recruiting into lymph nodes could prevent renal disease progression [94]. Severe proteinuria can stimulate TECs to synthesize mediators that recruit inflammatory chemokines, triggering lymphangiogenesis before renal fibrosis [96]. Interestingly, new injury-induced LVs were found near glomeruli with tuft adhesions [83], which might fit with the theory that urine filtrates incorrectly into interstitium in these areas [87]. In diabetic models, sustained inflammation and upregulation of VEGF-C cause dysregulated proliferation of disorganized LVs, accelerating kidney damage [97]. Indeed, SAR131675, a selective VEGFR-3 inhibitor, is reported as a promising therapy since it ameliorates renal fibrosis through inhibiting lipotoxicity-related LVs formation [98]. Notably, renal fibrosis may in turn act on the formation of LVs. Following UUO-induced injury, the powerful fibrogenic cytokines TGFβ1 and connective tissue growth factor (CTGF) have been proved to be participants in lymphangiogenesis by activating VEGF-C [93,99]. TGFβ1 is the central player of fibrosis and has been found to improve VEGF-C expression in tubule cells as well as macrophages [93]. Additionally, elevated CTGF expression was detected around new-brown LVs in patients with obstructive nephropathy and diabetic nephropathy, suggesting a possible involvement of CTGF in lymphangiogenesis [99]. Development of fibrosis in several models can be attenuated by reduction of CTGF [99,100,101] (Figure 3).

### 5.3. Permeability and Renal Fibrosis

According to the research by Reddy ST et al. [102], nanoformulations with a size of 100 nm or larger would be blocked by the pore of lymphatic endothelial membrane, indicating a similar pore size of LECs to that of glomerular endothelium. Unlike the restricted permeability of glomeruli caused by three membranes [103], lymphatic capillaries only have monolayer of endothelial cells and therefore allow the filtration of macromolecular substances. Impaired lymphatic barrier has been reported in several organs. The increased number of LVs and enhanced vessel integrity are of great significance in inhibiting matrix remodeling and immune cells accumulation in myocardial infarction, while the dysfunctional lymphatic system may contribute to progression of cardiac diseases [11]. Recent studies provided a leap forward in our understanding of the lymphatic network in kidney diseases. However, the question remains about how permeability of intrarenal LVs, which is the main feature of lymphatic capillaries, changes in renal fibrosis.

The first question is whether permeability of pre-existing LVs alters in the context of kidney fibrosis. Ultrastructural images of aged mesenteric LVs captured by electron microscopic scanning showed decreased glycocalyx and tight junction proteins, leading to damaged permeability as well as pathogen clearance. Reduced pumping function, which has been proved, also existed in nephrotic syndrome rats, combined with compromised endothelial cells barrier promoted the escape of immune cells in aged lymphatic collectors [104]. This lymphatic-related mechanism that explains the reduced ability of immune system in controlling infection in aging may similarly occur in renal fibrosis, which is also characterized by local as well as systemic immune dysfunction. Interestingly, after being induced by aging-like oxidative stress, cultured LECs had comparable permeability to the one treated with VEGF-A, a vascular permeability-associated factor [104]. VEGF-A-mediated pathological enlargement and hyperpermeability of cutaneous LVs contribute to compromised interstitial fluid clearance in the chronic response to injury [32]. VEGF-A expression was upregulated in diabetic kidney disease and strongly correlated with renal fibrosis [105]. Whether similar VEGF-A-associated leakage happens in the kidney is not yet known. Additionally, would injury-generated lymphatic capillaries have the same function as normal initial LVs, or some of them are just disorganized, non-functional vessels? During both embryological development and proliferation of LECs, zipper-like junctions firstly form between cells and subsequently differentiate into button-like ones [4]. This development pattern may lead to abnormal permeability of nascent lymphatic sprouts compared to normal initial LVs. Actually, it is of great significance to explore the conditions and timing for the formation of functional lymphatic capillaries since it may explain the contradictory effects of lymphangiogenesis on renal fibrosis in previous studies.

The effect of sodium on LVs in kidney diseases is another overlooked question. Kidney plays a major role in modulating sodium reabsorption as well as excretion, and various renal diseases are accompanied by sodium retention. We previously proved intrarenal sodium accumulation and elevated sodium concentration in renal lymph, causing the dysfunction of renal collecting LVs through Na-K-2Cl cotransporter NKCC1 [106]. Would accumulating interstitial sodium influence permeability of LVs or promote lymphangiogenesis through tonicity-related signaling such as cardiovascular disease? The involvement of tonicity-responsive enhancer-binding protein-induced VEGF-C expression and electrolyte clearance has been reported in hypertension mice [107]. Furthermore, it was recently proved that NO could not only influence contraction of LVs, but also compromise lymphatic vascular integrity. One unanticipated finding was that NO decreased permeability of type 2 diabetic LVs but increased permeability of non-diabetic, wild-type LVs [108]. Since renal fibrosis is also associated with NO deficiency [109], is NO also involved in barrier dysfunction of renal LVs in this context-dependent manner? For now, these questions remain unanswered and await further investigation.

### 5.4. Lymphatic-Related Treatment of Fibrosis

There are several LV-associated treatment strategies for chronic kidney injury that have been applied in studies (Table 2). VEGF-C 156S is a recombinant mutant form of VEGF-C in which Cys156 was replaced by Ser residue and referentially binds to VEGFR3 [110]. Continuous administration of VEGF-C 156S to UUO mice subsequently attenuated renal fibrosis by inducing lymphangiogenesis and decreasing level of immune cells in the interstitium. Recombinant human VEGF-C also reduced collagen I and TGF-β expression in UUO kidneys [95]. Transgenic technology for the specific expression of lymphopoietic factors is another strategy. Renal LVs hyperplasia in two hypertension models (salt-sensitive hypertension and NO synthase inhibition-induced hypertension) was enhanced by conditional overexpression of VEGF-D in kidney, with limited renal inflammatory cytokines accumulation before preventing hypertension [111]. However, to what extent VEGF-D overexpression may exert on renal fibrosis and remodeling is unclear.

Some studies identified lymphangiogenesis inhibitors as a therapeutic strategy to alleviate fibrosis. Separate suppression of two classical lymphangiogenic signaling pathways VEGF-C/VEGF-D–VEGFR-3 and FGF-2–LYVE-1 with soluble VEGFR-3 and LYVE-1 ameliorated renal inflammation and fibrosis [94]. Another study implemented anti-VEGFR-3 antibody, clodronate liposomes (block macrophages influx) or S1P agonist (block influx of lymphocyte and myofibroblast) to investigate the function and interaction of these three factors on tubulointerstitial fibrosis [112]. Interventions were administered to adriamycin-injected rats after the appearance of proteinuria, and results showed decreased lymphatic number in renal cortex without altering collagen accumulation or tubulointerstitial fibrosis. Although the authors proposed that renal fibrosis was not related to lymphatic proliferation in proteinuric situation, the relatively late start of treatment may have influenced the outcome. In another research, mouse model containing missense mutation in Vegfr3 did not exhibit change in renal function both in normal and low-dose cisplatin-mediated CKD [113]. However, they also proved cisplatin injury caused increased lymphatic density in renal cortex was due to decreased cortex volume, not due to changes in lymphatic volume or length. Additionally, the result in this study was based on low-dose cisplatin and a relatively short intervention time (4 weeks), leading to a mild renal injury and inconspicuous histologic findings. Therefore, in future investigations, it might be possible to perform with later time points and more severe injury.

### 5.5. The Lymphatic System: A New Pathway Mediating Crosstalk among Kidney and Other Organs

Kidney diseases are always associated with multiple organs dysfunction, which causes complications such as cardiovascular disease and dysregulated lipid metabolism [114]. Most early quantitative studies proved CKD produced toxic metabolites and disrupted intestinal barrier, none of these addressed intestinal lymphatic change in renal diseases. However, Zhong et al. recently suggested proteinuric kidney injury without renal failure stimulates lymphangiogenesis in intestine [115]. Enhanced oxidative stress and lipid peroxidation in kidney disease generated iso-levuglandin (IsoLG), a kind of lipid aldehydes. Only after the modification of IsoLG, apolipo-protein A1 directly increased lymphatic vasoconstriction, activated LEC and stimulated VEGF-C secretion from macrophages, thereby increasing intestinal lymph flow. Gut-originated harmful materials, such as IsoLG modified apolipo-protein A1, can be more easily transported throughout body by the increased gut lymphatic network induced by kidney injury, causing systemic dyslipidemia, which is the independent risk factor of cardiovascular diseases. Since cardiac and intestinal complications are more obvious in CKD, future studies should seek to evaluate the link among these three organs, with lymphatic-related explanations.

## 6. Conclusions

Notwithstanding the great progress that has been achieved toward understanding LVs in physiological and pathological processes, as highlighted in this article, considerably more work will need to be carried out on lymphatic capillaries. Interstitial substances are taken up by initial LVs via paracellular and transcellular pathways, and future studies exploring the factors influencing LV permeability may be a potential treatment for many diseases such as lymphoedema. Additionally, improved understanding of protective and pathogenic functions of proliferated LVs in a variety of disease processes, especially chronic kidney disease, is urgently needed. The change in permeability and proliferation of lymphatic capillaries in renal fibrosis remains understudied and new insights into lymphatic-mediated organs crosstalk are continuously developing. The development of new techniques, such as 3D reconstruction, offers the possibility to study the function of initial LVs in vivo in the future.

## Figures and Tables

**Table 1:** **Emerging technologies of lymphatic research**.

Method	Description	Tissues
Digital three-dimensional reconstruction	Combination of immuno-staining, optical clearance and confocal microscopy imaging to achieve three-dimensional images of isolated tissues	skin; kidney; heart
Fluorescent dyes with/without transgenic mice	Transgenic mice expressed mOrange, tdTomato or green fluorescent protein under Prox1 transcriptional control to better visualize lymphatic network	adrenal medulla; skin; cornea; tumor; lung, kidney, heart, diaphragm, intestine, mesentery, liver; ocular surface;
Cannulation	Cannulation of isolated lymphatic vessels can be used to study contractility of lymphatic vessels under multiple controlled experimental conditions	mesentery; heart; kidney

**Table 2:** **Lymphatic-related treatment of chronic kidney diseases.**

Strategy	Model	Mechanism and Dosage	Results
VEGF-C-156S	UUO	Recombinant VEGF-C (Cys156Ser) is a selective VEGF-C agonist 10 μg/100 g BW per day for 14 days; intraperitoneal injection	① Enhanced proliferation, not dilatation, of LVs ② Suppressed interstitial renal fibrosis; decreased collagen I level ③ Attenuated immune cells infiltration
LYVE-1-Cre/iDTR mice	UUO IRI	LYVE1^+^ LVs could be ablated in a DT-dependent manner 1.25 ng/g BW single dose; intravenous injection	① Attenuated lymphocyte expansion, perirenal lymphadenectasis, and splenomegaly ② Suppressed interstitial inflammatory infiltration and renal fibrosis
Soluble VEGFR-3 or LYVE-1 fusion constructs	UUO IRI	Inhibit lymphangiogenesis by suppressing VEGF-C/D-VEGFR-3 and FGF2-LYVE1 signaling pathway 0.5 μg/g BW 24 h before UUO or IRI; tail vein injection	① Decreased LV density in kidney and RDLN ② Ameliorated renal inflammation ③ Ameliorated renal fibrosis
KidVD^+^ mice	Salt-sensitive hypertension L-NAME–Induced hypertension	Transgenic mice with kidney specific overexpression of VEGF in doxycycline-dependent manner 1 week before L-NAME administration; continued L-NAME and doxycycline for 3 weeks; oral administration	① Augmented renal lymphangiogenesis and prevented development of hypertension ② Renal lymphatic expansion ③ Reduced immune cells accumulation
IMC-3C5	Adriamycin rats	Anti-VEGFR-3 antibody 40 mg/kg BW; 3 times/week for 6 weeks; intraperitoneal injection	① Prevented LVs formation ② Did not effect inflammatory, fibrotic level
*Vegfr3*Chy/^+^ mice	Cisplatin-mediated injury	Transgenic mice with abrogated kinase ability of *Vegfr3*	① No change in the magnitude of renal dysfunction ② Increased renal LVs density due to the loss of cortex

BW: body weight; IRI: ischemia reperfusion injury; L-NAME: nitro-l-arginine methyl ester hydrochloride; LV: lymphatic vessel; RDLN: renal draining lymph node; UUO: unilateral ureteral obstruction.

## Data Availability

Data sharing is not applicable to this article as no datasets were generated or analyzed during the current study.
